# *N*-Benzyl-2-(2-chloro-5-methyl­phen­oxy)acetamide

**DOI:** 10.1107/S1600536808038555

**Published:** 2008-11-22

**Authors:** Zhu-Bo Li, Xiao-Yan He, Yong-Sheng Xie, Guang-Cheng Qin

**Affiliations:** a College of Pharmaceutical Sciences, Southwest University, Chongqing 400716, People’s Republic of China; b School of Chemistry and Chemical Engineering, Shandong University, Jinan 250100, People’s Republic of China

## Abstract

The asymmetric unit of the title compound, C_16_H_16_ClNO_2_, contains two crystallographically independent mol­ecules, which differ mainly in the orientation of the benzyl group with respect to the rest of the mol­ecule. In the crystal packing, centrosymmetrically related mol­ecules are linked into dimers *via* inter­molecular C—H⋯O hydrogen-bond inter­actions.

## Related literature

For a related structure, see: Li *et al.* (2008 ▶). For hydrogen-bond motifs, see: Bernstein *et al.* (1995 ▶).
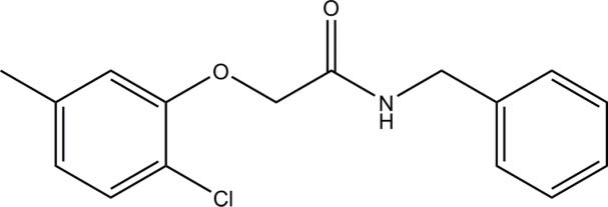


## Experimental

### 

#### Crystal data

C_16_H_16_ClNO_2_
*M* *_r_* = 289.75
Triclinic, 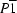

*a* = 9.5549 (18) Å
*b* = 11.086 (2) Å
*c* = 14.725 (3) Å
α = 71.747 (4)°
β = 89.062 (4)°
γ = 85.655 (4)°
*V* = 1477.0 (5) Å^3^
*Z* = 4
Mo *K*α radiation
μ = 0.26 mm^−1^
*T* = 298 (2) K
0.12 × 0.10 × 0.06 mm

#### Data collection

Bruker SMART CCD area-detector diffractometer
Absorption correction: multi-scan (*SADABS*; Bruker, 2005 ▶) *T*_min_ = 0.970, *T*_max_ = 0.985
7851 measured reflections
5194 independent reflections
2351 reflections with *I* > 2σ(*I*)
*R*_int_ = 0.033

#### Refinement

*R*[*F* ^2^ > 2σ(*F* ^2^)] = 0.054
*wR*(*F* ^2^) = 0.153
*S* = 0.98
5194 reflections
362 parameters
H-atom parameters constrained
Δρ_max_ = 0.18 e Å^−3^
Δρ_min_ = −0.23 e Å^−3^

### 

Data collection: *SMART* (Bruker, 2005 ▶); cell refinement: *SAINT* (Bruker, 2005 ▶); data reduction: *SAINT*; program(s) used to solve structure: *SHELXS97* (Sheldrick, 2008 ▶); program(s) used to refine structure: *SHELXL97* (Sheldrick, 2008 ▶); molecular graphics: *XP* in *SHELXTL* (Sheldrick, 2008 ▶); software used to prepare material for publication: *SHELXL97*.

## Supplementary Material

Crystal structure: contains datablocks I, global. DOI: 10.1107/S1600536808038555/rz2267sup1.cif

Structure factors: contains datablocks I. DOI: 10.1107/S1600536808038555/rz2267Isup2.hkl

Additional supplementary materials:  crystallographic information; 3D view; checkCIF report

## Figures and Tables

**Table 1:** Hydrogen-bond geometry (Å, °)

*D*—H⋯*A*	*D*—H	H⋯*A*	*D*⋯*A*	*D*—H⋯*A*
C3—H3⋯O4^i^	0.93	2.51	3.423 (4)	169
C19—H19⋯O2^i^	0.93	2.37	3.287 (4)	169

Symmetry code: (i) 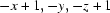.

**Fig. 1.**
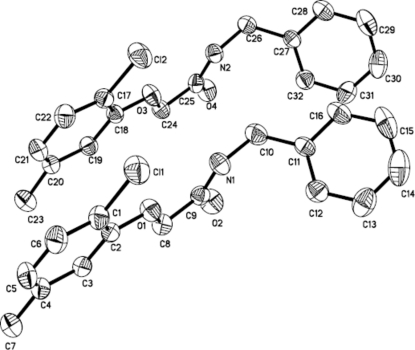
The molecular structure of the title compound, showing displacement ellipsoids drawn at the 50% probability level for non-H atoms.

## supplementary crystallographic information

### Comment

As part of our continuing project on the study of the interactions occurring between small molecules and proteins (Li *et al.*; 2008), we report here the synthesis and crystal structure of the title compound.

The asymmetric unit of the title compound (Fig. 1) contains two crystallographically independent molecules which differ mainly in the orientation of the benzyl groups (C10—C15 and C26—C32) bound to the amidic N atoms with respect of the mean plane through the rest of the molecule (dihedral angles of 87.03 (8) and 68.74 (6)° respectively). The O1—C8—C9—O2 and O3—C24—C25—O4 torsion angles are -176.5 (3) and -179.0 (3)°, respectively. These values can be compared with that of 10.5 (3)° found in the similar dichloro compound (Li *et al.*; 2008). In the crystal structure, centrosymmetrically related molecules are linked into dimers by intermolecular C—H···O hydrogen bonds (Table 1), forming fourteen-membered rings of graph set motif *R*^2^_2_(14) (Bernstein *et al.*, 1995).

### Experimental

A solution of 2-chloro-5-methylphenol (1.0 mmol), *N*-benzyl-2-chloroacetamide (1.1 mmol), K_2_CO_3_ (1.1 mmol) and CH_3_CN (20 ml) was refluxed for 3 h. After completion of the reaction (by TLC monitoring), the solution was cooled and the solvent was evaporated under reduced pressure. The residue was poured into water and adjusted to pH 6–7 with dilute hydrochloric acid (10%) and extracted with ethyl acetate, washed with brine and dried over anhydrous MgSO_4_. The mixture was then filtered and the filtrate obtained was concentrated under reduced pressure to obtain the corresponding crude product. The product was purified by column chromatography on silica gel using ethyl acetate as eluent (yield 86%). Crystals suitable for X-ray diffraction were obtained by slow evaparation of an ethyl acetate/hexane (1:1 v/v) solution at room temperature for 10 days.

### Refinement

All H atoms were placed in calculated positions and refined as riding, with C—H = 0.93-0.97 Å, N—H = 0.86 Å and with U_iso_(H) = 1.2U_eq_(C, N) or 1.5U_eq_(C) for methyl H atoms.

### Crystal data

**Table:**

C_16_H_16_ClNO_2_	*Z* = 4
*M**_r_* = 289.75	*F*_000_ = 608
Triclinic, *P*1	*D*_x_ = 1.303 Mg m^−^^3^
Hall symbol: -P 1	Mo *K*α radiation λ = 0.71073 Å
*a* = 9.5549 (18) Å	Cell parameters from 818 reflections
*b* = 11.086 (2) Å	θ = 5.5–38.5º
*c* = 14.725 (3) Å	µ = 0.26 mm^−^^1^
α = 71.747 (4)º	*T* = 298 (2) K
β = 89.062 (4)º	Block, colourless
γ = 85.655 (4)º	0.12 × 0.10 × 0.06 mm
*V* = 1477.0 (5) Å^3^	

### Data collection

**Table:**

Bruker SMART CCD area-detector diffractometer	5194 independent reflections
Radiation source: fine-focus sealed tube	2351 reflections with *I* > 2σ(*I*)
Monochromator: graphite	*R*_int_ = 0.033
*T* = 298(2) K	θ_max_ = 25.1º
φ and ω scans	θ_min_ = 1.5º
Absorption correction: multi-scan(SADABS; Bruker, 2005)	*h* = −11→11
*T*_min_ = 0.970, *T*_max_ = 0.985	*k* = −12→13
7851 measured reflections	*l* = −14→17

### Refinement

**Table:**

Refinement on *F*^2^	Hydrogen site location: inferred from neighbouring sites
Least-squares matrix: full	H-atom parameters constrained
*R*[*F*^2^ > 2σ(*F*^2^)] = 0.054	*w* = 1/[σ^2^(*F*_o_^2^) + (0.0578*P*)^2^] where *P* = (*F*_o_^2^ + 2*F*_c_^2^)/3
*wR*(*F*^2^) = 0.153	(Δ/σ)_max_ < 0.001
*S* = 0.98	Δρ_max_ = 0.18 e Å^−^^3^
5194 reflections	Δρ_min_ = −0.23 e Å^−^^3^
362 parameters	Extinction correction: SHELXL97 (Sheldrick, 2008), Fc^*^=kFc[1+0.001xFc^2^λ^3^/sin(2θ)]^-1/4^
Primary atom site location: structure-invariant direct methods	Extinction coefficient: 0.0039 (10)
Secondary atom site location: difference Fourier map	

### Special details

**Table:**

Geometry. All e.s.d.'s (except the e.s.d. in the dihedral angle between two l.s. planes) are estimated using the full covariance matrix. The cell e.s.d.'s are taken into account individually in the estimation of e.s.d.'s in distances, angles and torsion angles; correlations between e.s.d.'s in cell parameters are only used when they are defined by crystal symmetry. An approximate (isotropic) treatment of cell e.s.d.'s is used for estimating e.s.d.'s involving l.s. planes.
Refinement. Refinement of *F*^2^ against ALL reflections. The weighted *R*-factor *wR* and goodness of fit *S* are based on *F*^2^, conventional *R*-factors *R* are based on *F*, with *F* set to zero for negative *F*^2^. The threshold expression of *F*^2^ > σ(*F*^2^) is used only for calculating *R*-factors(gt) *etc*. and is not relevant to the choice of reflections for refinement. *R*-factors based on *F*^2^ are statistically about twice as large as those based on *F*, and *R*- factors based on ALL data will be even larger.

### Fractional atomic coordinates and isotropic or equivalent isotropic displacement parameters (Å^2^)

**Table:**

	*x*	*y*	*z*	*U*_iso_*/*U*_eq_	
Cl1	0.96319 (12)	0.34345 (9)	0.13691 (6)	0.0838 (4)	
Cl2	0.50957 (12)	0.36619 (10)	0.12091 (6)	0.0843 (4)	
O1	0.8583 (2)	0.1676 (2)	0.30620 (15)	0.0613 (7)	
O2	0.6330 (3)	−0.0799 (2)	0.39861 (18)	0.0816 (8)	
O3	0.3863 (2)	0.1968 (2)	0.28614 (14)	0.0612 (7)	
O4	0.1458 (3)	−0.0409 (2)	0.37055 (17)	0.0717 (8)	
N1	0.6903 (3)	0.0326 (3)	0.2484 (2)	0.0632 (8)	
H1	0.7406	0.0909	0.2139	0.076*	
N2	0.2154 (3)	0.0694 (2)	0.22282 (19)	0.0562 (8)	
H2	0.2722	0.1231	0.1905	0.067*	
C1	1.0023 (4)	0.3375 (3)	0.2529 (2)	0.0595 (10)	
C2	0.9435 (3)	0.2484 (3)	0.3297 (2)	0.0513 (9)	
C3	0.9725 (3)	0.2452 (3)	0.4215 (2)	0.0558 (9)	
H3	0.9333	0.1851	0.4726	0.067*	
C4	1.0594 (4)	0.3303 (4)	0.4391 (3)	0.0622 (10)	
C5	1.1172 (4)	0.4175 (4)	0.3616 (3)	0.0755 (12)	
H5	1.1763	0.4749	0.3720	0.091*	
C6	1.0892 (4)	0.4211 (3)	0.2698 (3)	0.0739 (11)	
H6	1.1293	0.4805	0.2187	0.089*	
C7	1.0907 (4)	0.3245 (4)	0.5403 (3)	0.0881 (13)	
H7A	1.0174	0.2841	0.5814	0.132*	
H7B	1.0959	0.4093	0.5433	0.132*	
H7C	1.1787	0.2763	0.5605	0.132*	
C8	0.7986 (3)	0.0726 (3)	0.3822 (2)	0.0582 (10)	
H8A	0.7484	0.1121	0.4246	0.070*	
H8B	0.8726	0.0135	0.4189	0.070*	
C9	0.6998 (4)	0.0018 (3)	0.3432 (3)	0.0553 (9)	
C10	0.5997 (4)	−0.0276 (4)	0.2010 (3)	0.0732 (11)	
H10A	0.5485	0.0379	0.1504	0.088*	
H10B	0.5316	−0.0715	0.2466	0.088*	
C11	0.6762 (4)	−0.1216 (3)	0.1586 (2)	0.0555 (9)	
C12	0.8057 (4)	−0.1810 (4)	0.1908 (3)	0.0680 (11)	
H12	0.8494	−0.1633	0.2407	0.082*	
C13	0.8722 (5)	−0.2667 (4)	0.1503 (4)	0.0905 (14)	
H13	0.9598	−0.3059	0.1731	0.109*	
C14	0.8103 (7)	−0.2939 (5)	0.0774 (4)	0.1025 (17)	
H14	0.8551	−0.3511	0.0498	0.123*	
C15	0.6804 (7)	−0.2356 (5)	0.0448 (3)	0.0996 (17)	
H15	0.6371	−0.2539	−0.0049	0.119*	
C16	0.6137 (4)	−0.1502 (4)	0.0853 (3)	0.0738 (12)	
H16	0.5257	−0.1118	0.0627	0.089*	
C17	0.5350 (4)	0.3631 (3)	0.2384 (2)	0.0540 (9)	
C18	0.4702 (3)	0.2756 (3)	0.3119 (2)	0.0496 (9)	
C19	0.4939 (3)	0.2724 (3)	0.4050 (2)	0.0540 (9)	
H19	0.4503	0.2141	0.4549	0.065*	
C20	0.5820 (4)	0.3550 (3)	0.4249 (2)	0.0556 (9)	
C21	0.6439 (4)	0.4419 (3)	0.3503 (3)	0.0657 (10)	
H21	0.7020	0.4986	0.3627	0.079*	
C22	0.6209 (4)	0.4461 (3)	0.2575 (3)	0.0663 (10)	
H22	0.6636	0.5052	0.2076	0.080*	
C23	0.6128 (4)	0.3443 (4)	0.5272 (2)	0.0848 (13)	
H23A	0.7020	0.2976	0.5462	0.127*	
H23B	0.5407	0.3005	0.5677	0.127*	
H23C	0.6155	0.4280	0.5330	0.127*	
C24	0.3147 (3)	0.1067 (3)	0.3610 (2)	0.0556 (9)	
H24A	0.2613	0.1507	0.3993	0.067*	
H24B	0.3826	0.0454	0.4024	0.067*	
C25	0.2180 (4)	0.0391 (3)	0.3175 (3)	0.0551 (9)	
C26	0.1215 (4)	0.0163 (3)	0.1709 (2)	0.0634 (10)	
H26A	0.0899	0.0824	0.1128	0.076*	
H26B	0.0395	−0.0100	0.2098	0.076*	
C27	0.1878 (3)	−0.0956 (3)	0.1448 (2)	0.0514 (9)	
C28	0.1796 (4)	−0.1007 (4)	0.0525 (3)	0.0666 (11)	
H28	0.1372	−0.0315	0.0052	0.080*	
C29	0.2332 (5)	−0.2062 (5)	0.0292 (3)	0.0821 (13)	
H29	0.2261	−0.2079	−0.0333	0.099*	
C30	0.2966 (5)	−0.3083 (4)	0.0978 (4)	0.0830 (13)	
H30	0.3314	−0.3800	0.0824	0.100*	
C31	0.3087 (4)	−0.3043 (4)	0.1899 (3)	0.0763 (12)	
H31	0.3548	−0.3721	0.2363	0.092*	
C32	0.2528 (4)	−0.2002 (4)	0.2128 (3)	0.0654 (10)	
H32	0.2586	−0.1998	0.2757	0.078*	

### Atomic displacement parameters (Å^2^)

**Table:**

	*U*^11^	*U*^22^	*U*^33^	*U*^12^	*U*^13^	*U*^23^
Cl1	0.1148 (9)	0.0760 (7)	0.0597 (6)	−0.0167 (6)	0.0051 (6)	−0.0180 (5)
Cl2	0.1155 (9)	0.0892 (8)	0.0509 (6)	−0.0252 (7)	0.0039 (6)	−0.0218 (5)
O1	0.0719 (17)	0.0588 (15)	0.0535 (14)	−0.0184 (14)	0.0021 (13)	−0.0150 (12)
O2	0.082 (2)	0.0840 (19)	0.0773 (18)	−0.0361 (17)	0.0169 (15)	−0.0170 (15)
O3	0.0689 (16)	0.0691 (16)	0.0527 (14)	−0.0265 (14)	0.0068 (12)	−0.0246 (12)
O4	0.0743 (18)	0.0733 (17)	0.0716 (17)	−0.0294 (15)	0.0172 (14)	−0.0235 (14)
N1	0.062 (2)	0.072 (2)	0.060 (2)	−0.0189 (17)	0.0046 (16)	−0.0251 (16)
N2	0.0544 (19)	0.0574 (19)	0.0608 (19)	−0.0111 (15)	0.0006 (15)	−0.0224 (15)
C1	0.070 (3)	0.055 (2)	0.056 (2)	−0.006 (2)	0.007 (2)	−0.0213 (19)
C2	0.048 (2)	0.045 (2)	0.062 (2)	0.0001 (18)	−0.0048 (19)	−0.0204 (18)
C3	0.058 (2)	0.053 (2)	0.059 (2)	0.0026 (19)	−0.0026 (19)	−0.0226 (18)
C4	0.062 (3)	0.059 (2)	0.074 (3)	0.004 (2)	−0.008 (2)	−0.033 (2)
C5	0.082 (3)	0.061 (3)	0.095 (3)	−0.016 (2)	−0.008 (3)	−0.039 (2)
C6	0.082 (3)	0.056 (3)	0.086 (3)	−0.016 (2)	0.005 (2)	−0.023 (2)
C7	0.100 (3)	0.086 (3)	0.091 (3)	0.003 (3)	−0.027 (3)	−0.047 (2)
C8	0.059 (2)	0.058 (2)	0.057 (2)	−0.008 (2)	0.0072 (19)	−0.0167 (18)
C9	0.048 (2)	0.057 (2)	0.062 (2)	−0.0025 (19)	0.002 (2)	−0.0193 (19)
C10	0.061 (3)	0.084 (3)	0.081 (3)	−0.009 (2)	−0.010 (2)	−0.034 (2)
C11	0.053 (2)	0.063 (2)	0.051 (2)	−0.017 (2)	−0.0016 (19)	−0.0167 (18)
C12	0.058 (3)	0.073 (3)	0.074 (3)	−0.012 (2)	0.000 (2)	−0.024 (2)
C13	0.082 (3)	0.068 (3)	0.125 (4)	−0.012 (3)	0.017 (3)	−0.035 (3)
C14	0.130 (5)	0.082 (4)	0.113 (4)	−0.041 (4)	0.055 (4)	−0.051 (3)
C15	0.150 (5)	0.094 (4)	0.074 (3)	−0.054 (4)	0.016 (4)	−0.044 (3)
C16	0.082 (3)	0.078 (3)	0.062 (2)	−0.027 (2)	−0.011 (2)	−0.017 (2)
C17	0.059 (2)	0.055 (2)	0.049 (2)	−0.0040 (19)	0.0014 (18)	−0.0158 (18)
C18	0.046 (2)	0.052 (2)	0.056 (2)	−0.0069 (18)	−0.0008 (18)	−0.0223 (18)
C19	0.057 (2)	0.056 (2)	0.050 (2)	−0.0069 (19)	0.0021 (18)	−0.0176 (17)
C20	0.061 (2)	0.054 (2)	0.056 (2)	−0.001 (2)	−0.0069 (19)	−0.0225 (18)
C21	0.069 (3)	0.065 (3)	0.072 (3)	−0.015 (2)	−0.007 (2)	−0.031 (2)
C22	0.072 (3)	0.058 (2)	0.069 (3)	−0.013 (2)	0.009 (2)	−0.019 (2)
C23	0.098 (3)	0.089 (3)	0.074 (3)	−0.005 (3)	−0.020 (2)	−0.034 (2)
C24	0.056 (2)	0.063 (2)	0.051 (2)	−0.0128 (19)	0.0041 (18)	−0.0208 (18)
C25	0.056 (2)	0.055 (2)	0.060 (2)	−0.003 (2)	0.008 (2)	−0.0263 (19)
C26	0.057 (2)	0.067 (3)	0.070 (2)	−0.004 (2)	−0.011 (2)	−0.027 (2)
C27	0.048 (2)	0.058 (2)	0.051 (2)	−0.0118 (19)	0.0000 (18)	−0.0193 (19)
C28	0.066 (3)	0.080 (3)	0.056 (2)	−0.021 (2)	0.000 (2)	−0.021 (2)
C29	0.092 (3)	0.105 (4)	0.066 (3)	−0.039 (3)	0.020 (3)	−0.044 (3)
C30	0.089 (3)	0.070 (3)	0.104 (4)	−0.028 (3)	0.034 (3)	−0.045 (3)
C31	0.085 (3)	0.055 (3)	0.086 (3)	−0.003 (2)	0.009 (3)	−0.018 (2)
C32	0.075 (3)	0.063 (3)	0.059 (2)	−0.007 (2)	0.000 (2)	−0.020 (2)

### Geometric parameters (Å, °)

**Table:**

Cl1—C1	1.735 (3)	C13—C14	1.357 (6)
Cl2—C17	1.741 (3)	C13—H13	0.9300
O1—C2	1.378 (3)	C14—C15	1.376 (6)
O1—C8	1.421 (3)	C14—H14	0.9300
O2—C9	1.225 (4)	C15—C16	1.380 (6)
O3—C18	1.369 (3)	C15—H15	0.9300
O3—C24	1.439 (3)	C16—H16	0.9300
O4—C25	1.231 (4)	C17—C22	1.372 (4)
N1—C9	1.332 (4)	C17—C18	1.381 (4)
N1—C10	1.442 (4)	C18—C19	1.381 (4)
N1—H1	0.8600	C19—C20	1.389 (4)
N2—C25	1.328 (4)	C19—H19	0.9300
N2—C26	1.456 (4)	C20—C21	1.373 (4)
N2—H2	0.8600	C20—C23	1.506 (4)
C1—C6	1.370 (5)	C21—C22	1.373 (4)
C1—C2	1.391 (4)	C21—H21	0.9300
C2—C3	1.373 (4)	C22—H22	0.9300
C3—C4	1.388 (4)	C23—H23A	0.9600
C3—H3	0.9300	C23—H23B	0.9600
C4—C5	1.383 (5)	C23—H23C	0.9600
C4—C7	1.504 (5)	C24—C25	1.500 (4)
C5—C6	1.371 (5)	C24—H24A	0.9700
C5—H5	0.9300	C24—H24B	0.9700
C6—H6	0.9300	C26—C27	1.504 (4)
C7—H7A	0.9600	C26—H26A	0.9700
C7—H7B	0.9600	C26—H26B	0.9700
C7—H7C	0.9600	C27—C28	1.382 (4)
C8—C9	1.500 (4)	C27—C32	1.384 (4)
C8—H8A	0.9700	C28—C29	1.380 (5)
C8—H8B	0.9700	C28—H28	0.9300
C10—C11	1.513 (5)	C29—C30	1.367 (6)
C10—H10A	0.9700	C29—H29	0.9300
C10—H10B	0.9700	C30—C31	1.377 (5)
C11—C16	1.374 (4)	C30—H30	0.9300
C11—C12	1.375 (5)	C31—C32	1.370 (5)
C12—C13	1.384 (5)	C31—H31	0.9300
C12—H12	0.9300	C32—H32	0.9300
			
C2—O1—C8	117.8 (2)	C16—C15—H15	119.7
C18—O3—C24	117.7 (2)	C11—C16—C15	120.5 (4)
C9—N1—C10	122.9 (3)	C11—C16—H16	119.7
C9—N1—H1	118.5	C15—C16—H16	119.7
C10—N1—H1	118.5	C22—C17—C18	120.5 (3)
C25—N2—C26	123.5 (3)	C22—C17—Cl2	119.8 (3)
C25—N2—H2	118.3	C18—C17—Cl2	119.7 (3)
C26—N2—H2	118.3	O3—C18—C19	124.6 (3)
C6—C1—C2	119.5 (3)	O3—C18—C17	116.4 (3)
C6—C1—Cl1	120.6 (3)	C19—C18—C17	119.0 (3)
C2—C1—Cl1	119.9 (3)	C18—C19—C20	120.8 (3)
C3—C2—O1	124.6 (3)	C18—C19—H19	119.6
C3—C2—C1	119.9 (3)	C20—C19—H19	119.6
O1—C2—C1	115.6 (3)	C21—C20—C19	118.9 (3)
C2—C3—C4	121.0 (3)	C21—C20—C23	121.3 (3)
C2—C3—H3	119.5	C19—C20—C23	119.7 (3)
C4—C3—H3	119.5	C22—C21—C20	120.8 (3)
C5—C4—C3	118.1 (3)	C22—C21—H21	119.6
C5—C4—C7	121.8 (3)	C20—C21—H21	119.6
C3—C4—C7	120.1 (3)	C17—C22—C21	120.0 (3)
C6—C5—C4	121.3 (3)	C17—C22—H22	120.0
C6—C5—H5	119.3	C21—C22—H22	120.0
C4—C5—H5	119.3	C20—C23—H23A	109.5
C1—C6—C5	120.2 (3)	C20—C23—H23B	109.5
C1—C6—H6	119.9	H23A—C23—H23B	109.5
C5—C6—H6	119.9	C20—C23—H23C	109.5
C4—C7—H7A	109.5	H23A—C23—H23C	109.5
C4—C7—H7B	109.5	H23B—C23—H23C	109.5
H7A—C7—H7B	109.5	O3—C24—C25	109.4 (3)
C4—C7—H7C	109.5	O3—C24—H24A	109.8
H7A—C7—H7C	109.5	C25—C24—H24A	109.8
H7B—C7—H7C	109.5	O3—C24—H24B	109.8
O1—C8—C9	110.1 (3)	C25—C24—H24B	109.8
O1—C8—H8A	109.6	H24A—C24—H24B	108.3
C9—C8—H8A	109.6	O4—C25—N2	123.4 (3)
O1—C8—H8B	109.6	O4—C25—C24	118.9 (3)
C9—C8—H8B	109.6	N2—C25—C24	117.7 (3)
H8A—C8—H8B	108.1	N2—C26—C27	113.6 (3)
O2—C9—N1	123.7 (3)	N2—C26—H26A	108.9
O2—C9—C8	119.4 (3)	C27—C26—H26A	108.9
N1—C9—C8	116.9 (3)	N2—C26—H26B	108.9
N1—C10—C11	114.0 (3)	C27—C26—H26B	108.9
N1—C10—H10A	108.7	H26A—C26—H26B	107.7
C11—C10—H10A	108.7	C28—C27—C32	117.4 (3)
N1—C10—H10B	108.7	C28—C27—C26	120.9 (3)
C11—C10—H10B	108.7	C32—C27—C26	121.7 (3)
H10A—C10—H10B	107.6	C29—C28—C27	121.3 (4)
C16—C11—C12	118.2 (4)	C29—C28—H28	119.4
C16—C11—C10	119.0 (4)	C27—C28—H28	119.4
C12—C11—C10	122.8 (3)	C30—C29—C28	120.1 (4)
C11—C12—C13	121.1 (4)	C30—C29—H29	120.0
C11—C12—H12	119.5	C28—C29—H29	120.0
C13—C12—H12	119.5	C29—C30—C31	119.6 (4)
C14—C13—C12	120.4 (5)	C29—C30—H30	120.2
C14—C13—H13	119.8	C31—C30—H30	120.2
C12—C13—H13	119.8	C32—C31—C30	119.8 (4)
C13—C14—C15	119.1 (5)	C32—C31—H31	120.1
C13—C14—H14	120.4	C30—C31—H31	120.1
C15—C14—H14	120.4	C31—C32—C27	121.7 (4)
C14—C15—C16	120.7 (5)	C31—C32—H32	119.1
C14—C15—H15	119.7	C27—C32—H32	119.1
			
C8—O1—C2—C3	−1.4 (5)	C24—O3—C18—C19	1.8 (5)
C8—O1—C2—C1	178.4 (3)	C24—O3—C18—C17	−178.5 (3)
C6—C1—C2—C3	0.2 (5)	C22—C17—C18—O3	179.9 (3)
Cl1—C1—C2—C3	−178.8 (3)	Cl2—C17—C18—O3	−1.1 (4)
C6—C1—C2—O1	−179.6 (3)	C22—C17—C18—C19	−0.4 (5)
Cl1—C1—C2—O1	1.4 (4)	Cl2—C17—C18—C19	178.5 (3)
O1—C2—C3—C4	−179.9 (3)	O3—C18—C19—C20	179.2 (3)
C1—C2—C3—C4	0.4 (5)	C17—C18—C19—C20	−0.4 (5)
C2—C3—C4—C5	−0.7 (5)	C18—C19—C20—C21	1.1 (5)
C2—C3—C4—C7	−179.5 (3)	C18—C19—C20—C23	−176.4 (3)
C3—C4—C5—C6	0.5 (6)	C19—C20—C21—C22	−1.0 (5)
C7—C4—C5—C6	179.3 (4)	C23—C20—C21—C22	176.4 (4)
C2—C1—C6—C5	−0.4 (6)	C18—C17—C22—C21	0.5 (6)
Cl1—C1—C6—C5	178.6 (3)	Cl2—C17—C22—C21	−178.5 (3)
C4—C5—C6—C1	0.0 (6)	C20—C21—C22—C17	0.2 (6)
C2—O1—C8—C9	175.0 (3)	C18—O3—C24—C25	174.4 (3)
C10—N1—C9—O2	0.1 (6)	C26—N2—C25—O4	3.4 (5)
C10—N1—C9—C8	180.0 (3)	C26—N2—C25—C24	−176.7 (3)
O1—C8—C9—O2	−176.5 (3)	O3—C24—C25—O4	−179.0 (3)
O1—C8—C9—N1	3.6 (4)	O3—C24—C25—N2	1.1 (4)
C9—N1—C10—C11	−105.2 (4)	C25—N2—C26—C27	−96.3 (4)
N1—C10—C11—C16	−157.2 (3)	N2—C26—C27—C28	−130.2 (3)
N1—C10—C11—C12	23.4 (5)	N2—C26—C27—C32	53.2 (4)
C16—C11—C12—C13	0.4 (5)	C32—C27—C28—C29	0.5 (5)
C10—C11—C12—C13	179.9 (3)	C26—C27—C28—C29	−176.2 (3)
C11—C12—C13—C14	0.0 (6)	C27—C28—C29—C30	−0.4 (6)
C12—C13—C14—C15	−0.4 (7)	C28—C29—C30—C31	−1.0 (6)
C13—C14—C15—C16	0.2 (7)	C29—C30—C31—C32	2.3 (6)
C12—C11—C16—C15	−0.6 (5)	C30—C31—C32—C27	−2.3 (6)
C10—C11—C16—C15	180.0 (3)	C28—C27—C32—C31	0.9 (5)
C14—C15—C16—C11	0.2 (6)	C26—C27—C32—C31	177.5 (3)

### Hydrogen-bond geometry (Å, °)

**Table:**

*D*—H···*A*	*D*—H	H···*A*	*D*···*A*	*D*—H···*A*
C3—H3···O4^i^	0.93	2.51	3.423 (4)	169
C19—H19···O2^i^	0.93	2.37	3.287 (4)	169

Symmetry codes: (i) −*x*+1, −*y*, −*z*+1.
